# Emotional Regulation Mechanisms of University Students in Group Work Situations

**DOI:** 10.3390/ejihpe14040058

**Published:** 2024-04-02

**Authors:** Lilyan Vega-Ramírez, Alda Reyno-Freundt, Christian Hederich-Martínez, Mª Alejandra Ávalos-Ramos

**Affiliations:** 1 EDUCAPHYS Research Group, Department of General and Specific Didactics, Faculty of Education, University of Alicante, 03690 Alicante, Spain; sandra.avalos@ua.es; 2 Department of Physical Activity Sciences, Faculty of Physical Activity and Sports Sciences, University of Playa Ancha, Valparaíso 08007, Chile; areyno@upla.cl; 3 Department of Education, Faculty of Social and Business Studies, University Autonoma of Manizales, Manizales 170003, Colombia; christian.hederichm@autonoma.edu.co

**Keywords:** motivation, abilities, collaborative work, social skills, higher education

## Abstract

Universities are active agents of social change through knowledge, providing citizens with the necessary abilities to face professional challenges. This work aims to evaluate and analyse the adaptation of emotional regulation in learning situations of group work in virtual and hybrid (virtual and presential) environments, of a group of students of Physical Activity and Sport Sciences belonging to a Chilean university and a Spanish university. Method: A total of 107 students from a Chilean university and a Spanish university, all of them enrolled in the degree in Physical Activity and Sport Sciences, participated in the study. The instrument used was the Adaptative Instrument for Regulation of Emotions questionnaire. Results: The analysis of the data shows that there are some significant differences (*p* ≤ 0.05), between the groups of students who worked virtually and those who worked in hybrid situations, in the aspects related to personal motivations (learn from my classmates, not to disappoint my working group, and enjoying the experience of working in a group). The students who worked online resolved conflicts mainly through individual regulation mechanisms with significant differences in relation to the students who worked in hybrid mode. No significant differences were found in the socioemotional challenges or in the balance of the metacognitive experience. Conclusion: The group that worked in hybrid learning valued group purposes over personal purposes and used the social regulation mechanism over individual regulation in conflict resolution. On the other hand, the group that worked virtually valued group and personal purposes equally and used the mechanism of individual regulation and social regulation to solve difficulties. Differences between students who worked in virtual and hybrid environments may be due to greater social interaction and group dynamics in hybrid environments, as well as differences in culture and access to resources and technology.

## 1. Introduction

One of the challenges of education in the 21st century is to respond to the social demands of today’s increasingly globalised world. Universities throughout history have constantly rethought their mission within their national and international environment to renew the institution as it is par excellence the generator of knowledge [1,2,3]. Therefore, Higher Education is seen as an element of social change that helps to boost the growth and development of countries [4,5].

From this perspective, the creation of the European Higher Education Area and the Bologna Process have brought about the most important changes faced by European universities. These have involved not only the equality of education, but also the transformation of education into active agents of social development through the creation of knowledge [6]. In these terms, educational institutions must provide citizens with the necessary competences to face new professional challenges [7]. Likewise, this fact is not alien to Higher Education institutions in Latin America. These institutions are forced to legitimise their mission in the face of the challenges of today’s society, demanding greater compliance in the generation of knowledge and their social responsibility [8], requiring a new approach based on the development of competences that respond to the demands of the labour market and scientific and technological development [9].

To respond to the learning competences established by the current Higher Education educational systems, teachers must be in permanent search of innovative methodological strategies that prioritise the development of students’ basic and professional competences [10]. To develop essential learning skills, active strategies such as project-based learning, flipped classrooms, and collaborative work, among others, are used. Through them, more autonomous and reflective learning could be achieved [11,12]. In this way, students can acquire the skills provided by the curricular content in an elaborate, organised and meaningful way, in order to solve possible situations in the professional and personal sphere [13].

Therefore, learning through active strategies delves into the student’s personal work such as collaborative work in the inquiry of information, which together should be discussed, developed, valued, and reworked, promoting different skills such as the solution to difficulties, negotiation, and time management [14]. In both educational and work environments, teamwork competence is one of the most valued qualities [15]. In this way, collaborative group work has become a pedagogical model and its implementation as a didactic strategy and resource has increased [16]. Teamwork can promote new actions that are supported by the exchange of knowledge and experiences among its participants. Basically, group work is based on a defined number of students working together on a task. To do so, they must distribute the tasks evenly, thus facilitating progress toward the established learning objectives [17,18].

Group work contains a diverse set of semi-structured teaching methodologies where students work together and support each other with their academic duties [19]. Consequently, students through a teaching–learning perspective based on positive interdependence can learn from and with their peers [20]. Therefore, in the approach to academic group work, there are needs associated with the organisation of tasks and groups that require creativity and autonomy, negotiations and agreements, and exchanges of information, and digital technologies can be used to facilitate these processes [21]. Several studies indicate that group work carried out over time would be a good procedure to expand self-knowledge, knowledge of the environment and teamwork, through the motivation of students [16,22,23,24], optimising learning and performance [18,25,26].

However, in the different situations of the teaching–learning process, the emotional dimension of group work is important in the motivation and self-regulating behaviour they demand [27]. Emotions in general could be seen as a basis for affective events that are generally triggered by internal or external stimuli [28]. Each person has a temperamental disposition that is affected by genetic components in addition to the environment and shared experiences with other people [29]. Emotional particularities could give rise to difficulties, causing the success or failure of a jointly elaborated academic work [30,31,32]. Enjoyment, boredom, and anger are emotions that could influence students’ task performance, as they could affect the motivation to participate in the proposed activity [33,34].

Throughout their evolutionary process, each subject adjusts their emotional regulation skills according to the specific needs of the stage they are going through [35]. Between the ages of 18 and 29, the beginning of adulthood, changes occur in personal and interpersonal relationships, with academic, work, and sexual aspects, as well as socioemotional status, being influential factors in these changes [35].

Furthermore, it is important to consider the access to technology in the field of education, which has facilitated and promoted virtual learning environments, generating changes in the way of being, knowing, acting, and relating to the main educational actors, teachers, and students. The use of technology constitutes a new way of transmitting knowledge, learning to learn, learning by doing, and establishing social relationships [36].

Therefore, our study aims to evaluate and analyse the adaptation of emotional regulation in group work learning situations in virtual and blended learning environments in a group of Physical Activity and Sport Science students from a Chilean university and a Spanish university.

## 2. Materials and Methods

The research had an exploratory approach, with a comparative post-test design, in which two groups of initial training in teaching Physical Activity and Sport in different locations (Spain and Chile) were compared. The group located in Spain worked on a theoretical subject in a blended learning format while the group located in Chile worked on a subject, also of a theoretical nature, in a completely virtual format. The research had an exploratory descriptive design, with a quantitative methodology.

### 2.1. Sample

The participants were 107 students (21 students from a Chilean university and 86 students from a Spanish university), all of them enrolled in the degree of Physical Activity and Sport Sciences in the academic year 2021/2022.

Students were informed that the data collected would be used for research purposes. Informed consent was obtained, following data protection guidelines and the approval of the UA-2020-11-22 Ethics Committee.

### 2.2. Instrument

This study used the AIRE (Adaptative Instrument for Regulation of Emotions) questionnaire developed by Järvenoja et al. [37]. This instrument assesses how emotions are regulated and adapted in different learning scenarios. The AIRE questionnaire aims to find out the problems and tensions that arise within the work team and the ways in which each subject deals with the emotions that result in interrelationships [37]. This questionnaire is composed of four blocks: the first deals with personal motivational goals, the second describes socio-emotional challenges, the third refers to emotional self-regulation mechanisms, and the last block inquires about the balance of individual and social metacognitive experience. This instrument was adapted by the Cognitive Style research group of the National Pedagogical University of Colombia [30].

### 2.3. Procedure

Through the university teaching research network in which faculty from two university institutions in Chile and Spain participate in the improvement of teaching, we proposed to analyse in depth the dynamics of learning and teaching strategies within this particular context. This will allow us to obtain more meaningful and applicable results for the continuous improvement of educational quality in both universities, as well as fostering closer and more effective collaboration between the researchers involved. Table 1 shows the main socio-demographic characteristics of the groups analysed.

Initially, the intention was for both groups of students to work in a semi-face-to-face environment. However, due to an unforeseen circumstance related to Chilean dynamics, which was not linked to the subject in question, the classes were moved to a fully online format. Therefore, of the two planned curricular activities, one was carried out virtually in Chile, while the other was carried out semi-presentially by the students in Spain. Once the process of the proposed activities was completed, the AIRE questionnaire was distributed through a Google Drive form for students to complete online in both groups. The questionnaire was available for a period of 10 days for completion.

The data were analysed using the statistical programme JASP^®^ 0.18.3 (Jefry Amazing Statistical Package). Descriptive statistics (percentages, cross-tabulations, mean, and standard deviation) were used for all variables. Given the non-compliance with the assumption of normality, the Mann–Whitney U statistic was used for the comparison of means, and the biserial rank correlation was used as the appropriate measure of effect size.

## 3. Results

We present the main results of the study, divided into the four dimensions contemplated by the authors of the instrument.

### 3.1. Personal Motivation

The most important personal objectives for the students related to group work are shown in Table 2. Of the thirteen objectives stated in the instrument, students valued firstly, taking responsibility for the work to be carried out, secondly, getting new ideas from the group work activities, and thirdly, learning as much as possible from their peers. Significant differences (≤0.005) were found in the evaluation of three suggested objectives (objectives 3, 6, and 8), with the Spanish students (hybrid), evaluating them more highly.

When asked about the two most important objectives from the above list, both Chileans (28.6%) and Spaniards (32.6%) highlighted in the first place, objective 3, “to learn as much as I can from my group mates”. In the second place, for Chilean students, a virtual learning environment, (28.6%), objective 8, was “to enjoy the group work experience as much as possible”, while for Spanish students, a hybrid learning environment, (23.8%), it was objective 4: “to get new ideas from the activities in my work group”. For both groups, objective 11 was the least important, “to make sure I don’t work harder than the rest of my group mates”.

### 3.2. Socio-Emotional Aspects

About the challenges and difficulties that arose, we noticed that the group work carried out by the students did not represent major difficulties. For both groups of university students, the main challenge was that some subjects presented excuses due to external circumstances, which prevented them from arriving on time or staying until the end of the meetings. For the Chilean students (virtual), there was also a secondary challenge concerning group members who were continually distracted, attending phone calls, or interrupting with topics that had nothing to do with work. On the other hand, for the Spanish students (hybrid), their secondary challenge was that their peers seemed to have very different styles of doing things. Some subjects in the group preferred to start work quickly, while others preferred to organise a work plan (Table 3).

### 3.3. Emotional Regulation

The emotional regulation mechanisms used by the students to solve the conflicts that arise are divided into (a) individual regulation mechanisms (IR), (b) co-regulation mechanisms (CO), and (c) social regulation mechanisms (SR). The mechanism of emotional regulation to resolve difficulties differs according to the group (Table 4). The Chileans who worked online resolved conflicts mainly by means of individual regulation mechanisms (the subject varies his/her way of appreciating problematic situations), with significant differences in relation to the students from Spain (hybrid). In turn, Chileans also use social regulation mechanisms (the whole group seeks a solution to the difficult scenario). On the other hand, the Spaniards who worked in blended learning mainly used the SR mechanism. Finally, the least used mechanism is CO (persuading peers).

### 3.4. Individual and Social Metacognitive Experience

Regarding the general assessment made by the students of the educational experience, and the achievement of the first two objectives indicated in block one of the questionnaires, we distinguish that approximately 80% of the students who worked online (Chileans) think that their goals were fully met. On the other hand, approximately 60% of the students who worked blended (Spaniards) think that the objectives were fully met, while 35% consider that they were relatively fulfilled. For a minority group (5%), the objectives were not achieved.

When asked about overall satisfaction with the work carried out, we found that approximately 75% of Chileans (online work) are totally satisfied, 14% are satisfied, and approximately 10% are moderately satisfied. On the other hand, approximately 50% of Spaniards (blended work) are totally satisfied, 40% are satisfied, 7% are moderately satisfied, and 3% are not satisfied.

## 4. Discussion

The purpose of this study was to evaluate and analyse the adaptation of emotional regulation in group work learning situations in virtual and blended learning environments in a group of Physical Activity and Sport Science students from a Chilean university and a Spanish university. The results of this study are relevant as they offer us a better understanding of the elements of social interaction and emotional regulation that occur during group work in different learning situations. This knowledge will be used to develop pedagogical strategies and resources to stimulate and foster more cooperative learning.

In this study, we found that students generally perceived group work as an enjoyable strategy and that it helped to develop certain social skills. The results showed that the main purposes and motivations of the students were to take responsibility for the work to be carried out, to obtain new ideas from the group work activities and to learn as much as possible from their peers. These aims would be in line with the specific purpose of group work, stated above, indicating that it offers an opportunity to exchange experiences and knowledge, where learners help each other, learning from and with their peers [17,18,19,20,25,26,38,39].

It is noteworthy that Spanish students with a blended learning situation reported higher ratings on learning-related goals, some of them with significant differences, on personal performance goals. These data are consistent with [37], who found that students studying in face-to-face settings reported significantly more learning goals and fewer performance goals than their peers in virtual groups, despite a similar overall goal orientation. This is evidence that student goals can be generated according to learning situations and contexts. In hybrid learning environments, where there is a combination of virtual and face-to-face interactions, students are likely to have more opportunities to develop stronger interpersonal relationships with their peers. This may influence the way they regulate emotions, prioritising group work over personal goals. In comparison, in virtual environments, interaction may be more limited, which could lead to a greater reliance on individual mechanisms of emotional regulation.

In a group learning situation, tensions and difficulties inevitably arise during group activities due to differences in their respective goals, priorities, and expectations [37,40], which can trigger different emotions. The main difficulty encountered by the students from both universities was that some of the group members had personal and family commitments that made it difficult for them to meet each other or made them leave the practice earlier. Under this premise, Järvenoja et al. [37] and Volet and Mansfield [41] refer to the fact that one of the problems that working groups may encounter is related to external circumstances, such as practical obstacles (e.g., public transport) or other commitments that may limit responsibility and full participation. Another aspect worth mentioning is that Chilean students were also challenged by the fact that their peers were constantly busy on the phone or dealing with issues that had nothing to do with the topic of the work.

In contrast, for the Spanish students, the challenge was that their peers seemed to have very different ways or styles of doing things. These data are also consistent with those reported by Burdett [42] and Järvenoja et al. [37], who argue that the achievement of personal goals is embedded in a multitude of possible distractions, which interfere with group work. In addition, different levels of commitment or concentration, or power relations between members may also create challenges that can affect the quality of teamwork. The group can also be affected by conflicts generated by different working styles or different ways of interacting and communicating [37,43].

As for the mechanisms of emotional regulation to solve the difficulties arising in the group learning process, there are different ways of channelling and regulating the emotions that arise in these scenarios: from the construction of narratives and internal rationalities (self-regulation), through different attempts at convincing (co-regulation), to the construction of group communicative scenarios (social regulation). Our results show differences in emotional regulation between the groups of students, with Chilean students using mainly the individual regulation mechanism with a significant difference with respect to Spanish students. These differences may be related to the way of approaching group work, as the Chilean participants worked online. Nowadays, technology allows communication, interaction, and knowledge exchange to happen effectively. Social relationships in virtual contexts require empathy among group members, i.e., the ability to understand and comprehend their peers [37]. The most manifest skills for individual regulation of Chilean students were the self-conviction of the benefit of understanding, reflection, and performance improvement, a background that agrees with Luptáková and Antala [44], who argue the importance of being flexible and having the conviction that the context is conducive and is elementary to cope with possible differences that may arise within the group.

On the other hand, our study showed that mainly Spanish students and, secondarily, Chilean participants used social regulation within the group, stating that they were committed to conciliating and fulfilling common goals. The ability to manage and organise tasks and establish group priorities was noted, a fact also observed by Herrera-Pavo [45], where students with similar skills, interests, and experiences tend to be better at achieving the objectives proposed by the group. Another issue to highlight about social engagement was the acceptance of decisions and opinions of others and the mediation of possible problems.

Regarding the individual and group metacognitive experience, the balance made by the students in relation to the fulfilment of objectives and the satisfaction of group work was mostly positive in both learning situations, with the results being better valued by the group of Chilean students, but without significant differences with the group of Spanish participants. It should be noted that these positive ratings may be related to the group goals set by the students over personal goals; on the contrary, the few negative ratings may reflect a tendency towards egocentrism where they see group tasks in terms of themselves within the group [41].

After analysing the results presented, we consider it important to make some reflections for university teaching, since group work is a resource widely used by initial teacher training. Learning through collaborative group activities, online, blended, and face-to-face, not only offers a metacognitive purpose, but also offers the possibility of promoting and developing socialisation, and building personal, professional, and intellectual knowledge [36]. This study highlights the importance of valuing the social learning processes of university students in different scenarios such as virtual and blended learning. These results provide detailed insights into how students perceive and are motivated to participate in group work, as well as differences in learning goals between different learning contexts. This provides valuable information for designing teaching and learning strategies that make the most of the potential of group work and adapt to the needs and preferences of students in different learning environments.

Increased self-efficacy, performance, and academic aptitude are concerned with students’ emotional regulation [4,46,47]. One of the aims of group work is to increase the competences that teach students to face both their personal and professional future. To achieve this purpose, it is necessary to create educational structures that are supported by the purpose of group work, which cements a collective knowledge where each member of the group is responsible for their own learning and that of others [14]. Taken together, these results highlight the complexity of working in groups and point to several factors that can affect team dynamics and performance. This underlines the importance of addressing these difficulties proactively and fostering communication, conflict resolution and teamwork skills among students to improve the effectiveness of group learning activities.

The limitations of this study refer to the differentiation between the groups compared in terms of country, institution, and cultural characteristics; we understand the significance of this aspect and how it may influence our results. It is essential to bear in mind that these contextual factors may introduce additional variability in our observations and analysis. Given the diversity of contexts among the groups studied, we believe that our comparisons should be interpreted with caution. Although we have made efforts to control for relevant variables, we recognise that these contextual differences may affect the generalisability of our findings. In addition, it is necessary to point out as a limitation of this study that we do not have a cross-culturally validated tool, although the instrument has been validated for the Colombian population.

With respect to the small sample size in one of the groups compared to the other group, it is crucial to note how these factors may influence the interpretation of our results. The small sample and lower enrolment may limit the generalisability of our findings and the robustness of our analyses, introducing potential biases and limiting the external validity of our results.

We hope to address these limitations in future research by exploring strategies to expand our samples and improve the robustness of our analyses.

## 5. Conclusions

After the evaluation and analysis of the adaptation of emotional regulation in group work learning situations, in virtual and blended learning environments, the following was found:

- The objectives or goals are similar in both learning situations, with group learning objectives being more highly valued than personal goals, with some significant differences in favour of the Spanish students who worked with blended learning.
- The socio-emotional challenges faced mainly by students were related to external factors such as family or personal commitments that limited participation. Other secondary challenges were related to distractions and different ways of working.
- The emotional regulation mechanisms used by the students are mainly for the Chilean group (virtual environments) individual regulation and social regulation. The group of Spanish participants (blended environment) mainly used social regulation.
- The general evaluation of the metacognitive experience in terms of the fulfilment of objectives and satisfaction with the work carried out was mostly positive in both groups.
- Group work, both online and blended, is a strategy that can develop competences that prepare students for both professional and personal life.
- The results detail how students view, feel motivated by and cope with the challenges of teamwork, as well as the differences in their learning goals in different educational contexts. They highlight the inherent complexity of teamwork and the need to actively address barriers by fostering skills such as effective communication, conflict resolution, and collaborative work to improve the effectiveness of group learning. Taken together, these results provide an in-depth insight into how students manage their emotions, regulate their behaviour and reflect on their learning process during group activities, taking into account cultural and contextual influences. This knowledge is essential for designing educational interventions that foster students’ holistic development in collaborative learning environments.
- The use of the AIRE instrument can contribute to students’ perception of the socio-emotional aspects of group learning activities in different learning situations, contributing to the regulation of shared learning processes for the achievement of goals.

## Figures and Tables

**Table 1:** Socio-demographic characteristics of the groups analysed.

Characteristics	Chile	Spain
Type of university	Public	Public
Location	Valparaíso is a port city on the coast of Chile. Number of inhabitants 295.113	Alicante is a port city on the Mediterranean coast. Number of inhabitants 331.577
Grade	Physical activity and sport sciences	Physical activity and sport sciences
Curriculum	42 subjects, 9 semesters	39 subjects, 8 semesters
Subject	Didactic (sixth semester)	Didactic (fifth semester)
Enrolment in the subject	36 students	112 students
Average age of students	22.71 ± 1.488	22.90 ± 3.004
Methodologies used	Lectures, individual and group work, active methodologies.	Lectures, individual and group work, active methodologies.
Contents	Teacher behaviour, curricular bases, organisation of learning objectives for different educational levels, differentiation of classroom styles, teaching styles	General introduction to the didactics of physical education and sport, teaching intervention and teaching strategies in physical education and sport, planning and programming in physical education and sport.

**Table 2:** Personal motivations for group work.

Objectives		N	*Mdn*	IQR	*u*	*p*	*r_b_*
1. Achieve the highest grade and stand out above the rest of my classmates.	Virtual	21	2.00	2.00	836.50	0.580	−0.07
	Hybrid	86	2.00	1.00			
2. To do everything possible so that my grade would not be affected because of the group.	Virtual	21	3.00	1.00	753.00	0.212	−0.17
	Hybrid	86	3.00	1.00			
3. To learn as much as I can from my classmates.	Virtual	21	3.00	0.00	382.00	0.001	−0.58
	Hybrid	86	4.00	1.00			
4. To obtain new ideas from the activities in my working group.	Virtual	21	3.00	0.00 *			
	Hybrid	86	4.00	1.00			
5. Do my best not to get stressed.	Virtual	21	3.00	0.00	783.00	0.300	−0.13
	Hybrid	86	3.00	1.00			
6. Not to disappoint my working group.	Virtual	21	3.00	0.00	435.00	0.001	−0.52
	Hybrid	86	4.00	1.00			
7. Avoid appearing incompetent in front of the group.	Virtual	21	3.00	1.00	779.00	0.307	−0.14
	Hybrid	86	3.00	2.00			
8. Enjoy the experience of group work as much as I can.	Virtual	21	3.00	0.00	586.50	0.006	−0.35
	Hybrid	86	3.00	1.00			
9. To make new friends and/or socialise with other students in my work group.	Virtual	21	3.00	1.00	848.00	0.640	−0.06
	Hybrid	86	3.00	1.00			
10. To take responsibility for the work to be carried out.	Virtual	21	3.00	0.00 *			
	Hybrid	86	4.00	1.00			
11. To make sure that I do not work harder than the rest of my classmates.	Virtual	21	2.00	1.00	844.50	0.616	−0.06
	Hybrid	86	2.00	1.00			
12. Make sure that all my group mates contribute equally.	Virtual	21	3.00	0.00	790.50	0.309	−0.12
	Hybrid	86	3.00	0.00			
13. To use group work to develop my leadership skills.	Virtual	21	3.00	1.00	989.50	0.466	−0.10
	Hybrid	86	3.00	1.00			

N = number of subjects; *Mdn* = medium; IQR = inter-quartile range; *u* = Mann–Whitney U; *p* = significance; *r_b_* = effect size. *: for objectives 4 and 10, there are no variations in the responses of the virtual sample, so no significance values for differences and effect size measures are presented.

**Table 3:** Main challenges and difficulties of group work.

Challenges and Difficulties		N	*Mdn*	IQR	*u*	*p*	*r_b_*
1. In our work group, the objectives were different.	Virtual	21	1.00	2.00	871.00	0.789	−0.04
	Hybrid	86	1.00	2.00			
2. In our group, we had different priorities.	Virtual	21	1.00	0.00	917.00	0.880	0.02
	Hybrid	86	1.00	0.00			
3. In our work group, everyone seemed to have very different ways or styles of doing things.	Virtual	21	1.00	1.00	749.00	0.200	−0.17
	Hybrid	86	2.00	2.00			
4. In our work group, everyone seemed to have different interaction styles.	Virtual	21	1.00	1.00	809.00	0.401	−010
	Hybrid	86	1.00	1.00			
5. In our group, some did not get along.	Virtual	21	1.00	0.00	861.00	0.652	−0.05
	Hybrid	86	1.00	0.00			
6. In our work group, some people were not fully committed to the work.	Virtual	21	1.00	2.00	923.00	0.869	0.02
	Hybrid	86	1.00	1.75			
7. In our work group, some people had different priorities for getting the job carried out.	Virtual	21	1.00	1.00	894.00	0.940	−0.09
	Hybrid	86	1.00	1.00			
8. In our work group, some people were too competitive and individualistic.	Virtual	21	1.00	0.00	908.50	0.949	−0.06
	Hybrid	86	1.00	0.00			
9. In our work group, some people were easily distracted.	Virtual	21	2.00	1.75	1068.50	0.150	0.18
	Hybrid	86	1.00	1.00			
10. In our work group, I noticed that each one had a different idea about what should be carried out.	Virtual	21	1.00	1.00	953.50	0.642	0.06
	Hybrid	86	1.00	1.00			
11. In our work group, some people had very different knowledge and mastery of the subject.	Virtual	21	2.00	1.00	1014.50	0.343	0.12
	Hybrid	86	1.00	1.00			
12. In our work group, some people had personal, family or other circumstances and commitments.	Virtual	21	2.00	0.00	935.50	0.793	0.04
	Hybrid	86	2.00	2.00			

N = number of subjects; *Mdn* = medium; IQR = inter-quartile range; *u* = Mann–Whitney U; *p* = significance; *r_b_* = effect size.

**Table 4:** Emotional regulation mechanisms according to working group.

	Item		N	*Mdn*	IQR	*u*	*p*	*r_b_*
IR	I convinced myself that the situation could be a good thing.	Virtual	21	4.00	2.00	1152.50	0.04	0.28
		Hybrid	86	3.00	2.00			
IR	I tried to be flexible with the differences presented in the group.	Virtual	21	4.00	2.00	1215.00	0.01	0.35
		Hybrid	86	3.00	2.00			
IR	I tried to understand that the other people were not trying to play hard to get, but rather had different goals.	Virtual	21	3.00	3.00	1157.00	0.04	0.28
		Hybrid	86	2.00	2.00			
IR	I tried to accept and consider that some people are more prepared to work than others.	Virtual	21	3.00	2.00	1101.50	0.11	0.22
		Hybrid	86	2.00	3.00			
IR	Total	Virtual	21	3.00	2.00	1214.00	0.01	0.34
		Hybrid	86	2.00	2.00			
CO	I tried to persuade others that we needed to accept, that some people are more prepared to work than other forms.	Virtual	21	2.00	2.00	1075.00	0.140	0.19
		Hybrid	86	1.00	1.00			
CO	I tried to persuade the group to be more flexible to find a solution to the conflict situation.	Virtual	21	3.00	3.00	1175.00	0.031	0.30
		Hybrid	86	2.00	2.00			
CO	I tried to explain to the people in my group that we needed to understand the different objectives to carry out the work.	Virtual	21	3.00	3.00	1126.00	0.073	0.25
		Hybrid	86	2.50	1.00			
CO	I tried to convince the people in my group that some were not just playing hard to get, but that it was just their way.	Virtual	21	2.00	2.00	1070.00	0.150	0.18
		Hybrid	86	1.00	2.00			
CO	Total	Virtual	21	2.00	2.00	1159.50	0.044	0.28
		Hybrid	86	2.00	1.00			
SR	We understood that we had to reconcile our objectives to be able to develop our work as a group.	Virtual	21	3.00	3.00	980.00	0.970	0.54
		Hybrid	86	4.00	2.00			
SR	We resolved the situation by agreeing that we would agree on which of all the objectives to leave as a work goal.	Virtual	21	4.00	3.00	917.00	0.911	0.02
		Hybrid	86	4.00	2.00			
SR	We decided that we had to put our points of view aside and focus on the objective of the work.	Virtual	21	4.00	3.00	991.00	0.482	−0.07
		Hybrid	86	3.00	2.00			
SR	We accept that everyone has different objectives, and we develop group work.	Virtual	21	4.00	3.00	1013.00	0.383	0.13
		Hybrid	86	3.00	2.00			
SR	Total	Virtual		4.00	3.00	966.50	0.620	0.07
		Hybrid		3.00	2.00			

IR = individual regulation; CO = co-regulation; SR = social regulation. N = number of subjects; *Mdn* = medium; IQR = inter-quartile range; *u* = Mann–Whitney U; *p* = significance; *r_b_* = effect size.

## Data Availability

The data presented in this study are available on request from the corresponding author.
